# Mammographic calcifications undergoing percutaneous biopsy: outcome in women with and without a personal history of breast cancer

**DOI:** 10.1007/s11547-022-01583-5

**Published:** 2023-01-04

**Authors:** Valeria Bertani, Nicole Berger, Matthias Eberhard, Kristina Lång, Martina Urbani, Manuela La Grassa, Luca Balestreri, Andreas Boss, Thomas Frauenfelder, Magda Marcon

**Affiliations:** 1 Department of Oncologic Radiation Therapy and Diagnostic Imaging, Centro Di Riferimento Oncologico, Via Franco Gallini, 2, 33081 Aviano, Italy; 2 Institute of Diagnostic and Interventional Radiology, University Hospital Zurich, Raemistrasse 100, 8091 Zurich, Switzerland; 3 Institute of Radiology, Spital Lachen, Oberdorfstrasse 41, 8853 Lachen, Switzerland; 4 Department of Translational Medicine, Diagnostic Radiology, Lund University, Skane University Hospital, Jan Waldenströms Gata 22, S-205 02 Malmö, Sweden

**Keywords:** Digital mammography, Microcalcifications, Breast cancer

## Abstract

**Purpose:**

To compare the positive predictive values (PPVs) of BI-RADS categories used to assess pure mammographic calcifications in women with and without a previous history of breast cancer (PHBC).

**Materials and methods:**

In this retrospective study, all consecutive pure mammographic calcifications (*n* = 320) undergoing a stereotactic biopsy between 2016 and 2018 were identified. Mammograms were evaluated in consensus by two radiologists according to BI-RADS and blinded to patient history and pathology results. Final pathologic results were used as the standard of reference. PPV of BI-RADS categories were compared between the two groups. Data were evaluated using standard statistics, Mann–Whitney *U* tests and Chi-square tests.

**Results:**

Two hundred sixty-eight patients (274 lesions, median age 54 years, inter-quartile range, 50–65 years) with a PHBC (*n* = 46) and without a PHBC (*n* = 222) were included. Overall PPVs were the following: BI-RADS 2, 0% (0 of 56); BI-RADS 3, 9.1% (1 of 11); BI-RADS 4a, 16.2% (6 of 37); BI-RADS 4b, 37.5% (48 of 128); BI-RADS 4c, 47.3% (18 of 38) and BI-RADS 5, 100% (4 of 4). The PPV of BI-RADS categories was similar in patients with and without a PHBC (*P* = .715). Calcifications were more often malignant in patients with a PHBC older than 10 years (47.3%, 9 of 19) compared to 1–2 years (25%, 1 of 4), 2–5 years (20%, 2 of 10) and 5–10 years (0%, of 13) from the first breast cancer (*P* = .005).

**Conclusion:**

PPV of mammographic calcifications is similar in women with or without PHBC when BI-RADS classification is strictly applied. A higher risk of malignancy was observed in patients with a PHBC longer than 10 years.

**Supplementary Information:**

The online version contains supplementary material available at 10.1007/s11547-022-01583-5.

## Introduction

Women surviving a first breast cancer remain at risk to develop a subsequent local or regional recurrence in the same breast or a new primary cancer in the contralateral breast. In this circumstance, an increased rate of distant metastases and breast cancer mortality has been observed [1, 2]. Based on the evidence that mammography in the screening as well as in the surveillance setting reduces breast cancer mortality, current surveillance guidelines recommend annual mammography to asymptomatic patients with a personal history of breast cancer [3–8].

Breast-conserving therapy instead of mastectomy has become the treatment of choice for early-stage breast cancer, given equivalent survival rates [9, 10]. However, due to the effect of the combination of post-surgical changes and radiotherapy, e.g., architectural distortion, skin thickening and dystrophic calcifications, image interpretation can be more challenging. In general, 2–3 years are necessary to achieve the stability of the post-treatment-induced modifications [11].

Calcifications represent about 30% of suspicious findings leading to recall in population-based screening [12]. Assessment of calcifications is commonly based on radiologist evaluation according to the Breast Imaging-Reporting and Data System (BI-RADS) [13]. Evaluation of morphology and distribution of calcifications along with their variations over time should ensure an accurate categorization of the calcifications, but in practice, differentiation of benign and malignant disease remains challenging with high interobserver variability and false-positive biopsy rates of up to 87% [14–16]. BI-RADS classification is based on the imaging evaluation and should be applied regardless of personal factors such as a personal history of breast cancer. Nevertheless, evaluation of calcifications may be biased when a personal history of breast cancer (PHBC) is known. Moreover, considering a higher cancer rate than in women without PHBC and an interval cancer rate up to 35%, a biopsy could be more often recommended also in case of calcifications at low risk of malignancy [17–19].

We hypothesized that, if BI-RADS categorization is strictly applied in the assessment of calcifications, no differences in the clinical outcome are present between patients with and without a PHBC. The purpose of this study is to compare the positive predictive values (PPVs) of the BI-RADS categories used to assess mammographic calcifications in women with and without a PHBC.

## Material and methods

The institutional review board approved this retrospective study. The requirement for written informed consent was waived.

### Study population

All consecutive calcifications for which a stereotactic biopsy was performed from June 1, 2016, through May 31, 2018, at the Department of Oncologic Radiation Therapy and Diagnostic Imaging (Centro di Riferimento Oncologic, Aviano) were identified from the institutional radiology–pathology database. The following inclusion criteria were applied: (1) availability of digital mammograms or magnification views preceding the biopsy, (2) asymptomatic patients with calcifications without associated masses, asymmetries or architectural distortion, (3) patients with a PHBC with a follow-up period of at least 12 months and a first cancer stage 0–III, (4) final pathology result from either stereotactic biopsy or subsequent surgical excision for all lesions with a biopsy-proven diagnosis of malignancy, uncertain malignant potential or radiologic–pathologic discordance. The following cases were excluded: (1) patients undergoing 6-month follow-up examination for probably benign findings (BI-RADS 3), (2) patients with known BRCA mutation, (3) benign diagnosis after stereotactic biopsy with lack of imaging follow-up of at least 24 months, (4) patients with known simultaneous breast cancer in the ipsilateral or contralateral breast. Final pathologic results were used as the standard of reference.

### Data collection

In women without a PHBC, the following data were collected: information regarding incident or prevalent screening, a personal history of percutaneous biopsy or surgical excision for other than malignant lesions was recorded. In patients with a PHBC, the following data were collected: time since the first breast cancer diagnosis; histologic type; cancer stage; surgical method; adjuvant radiation therapy; adjuvant chemotherapy or endocrine therapy. Information regarding menopausal status and family history, except in case of known BRCA mutation, was not always available and has therefore not been included in further analyses.

### Imaging evaluation

The craniocaudal/mediolateral oblique mammogram or the magnification view containing the calcifications was evaluated in consensus by two radiologists with each 9 years of experience in breast imaging (M.M. and N.B.). The radiologists were informed about the position of the calcifications, if calcifications were a new finding or increasing compared to prior mammograms or if no previous mammograms were available for comparison. In case of multiple groups of calcifications in the same quadrant, the distance from the nipple of calcifications under investigation was also provided. A table with detailed specifications for the calcification assessment according to BI-RADS was provided (Online Appendix), and the ACR BI-RADS Atlas was available with calcification examples with corresponding categorization. The morphology and distribution of the calcifications and the corresponding BI-RADS category were annotated. No patient-related clinical information and pathology outcomes were provided.

### Statistical analysis

Descriptive statistics were used to summarize the characteristics of the patients and the lesions. The Mann–Whitney *U* test was used to compare age of patients with and without a PHBC. Data concerning pathology at diagnosis for the two groups and data from patients with a PHBC were analyzed with Chi-square and post hoc comparison; Bonferroni correction was applied for evaluating significance (*P* < 0.05/10 = 0.005 and *P* < 0.05/8 = 0.006). The PPV for calcification morphology, distribution and BI-RADS classifications was calculated by dividing the number of cases of malignancy (invasive or ductal in situ cancer) by the total number of cases in each category. The Wilcoxon signed rank test was used to compare the PPVs of calcification morphology and distribution as well as the PPV of the BI-RADS categories in patients with and without history of breast cancer. The PPV of the dataset from the original read-out (PPV^1^) was calculated by dividing the number of cases of malignancy (invasive cancer or ductal carcinoma in situ (DCIS)) by the total number of cases which underwent biopsy. All statistical calculations were performed using SPSS software (SPSS, version 22.0; SPSS), and *P* < 0.05 was considered to indicate statistical significance except for the Bonferroni corrections.

## Results

### Patient characteristics and pathology results

**Fig. 1:**
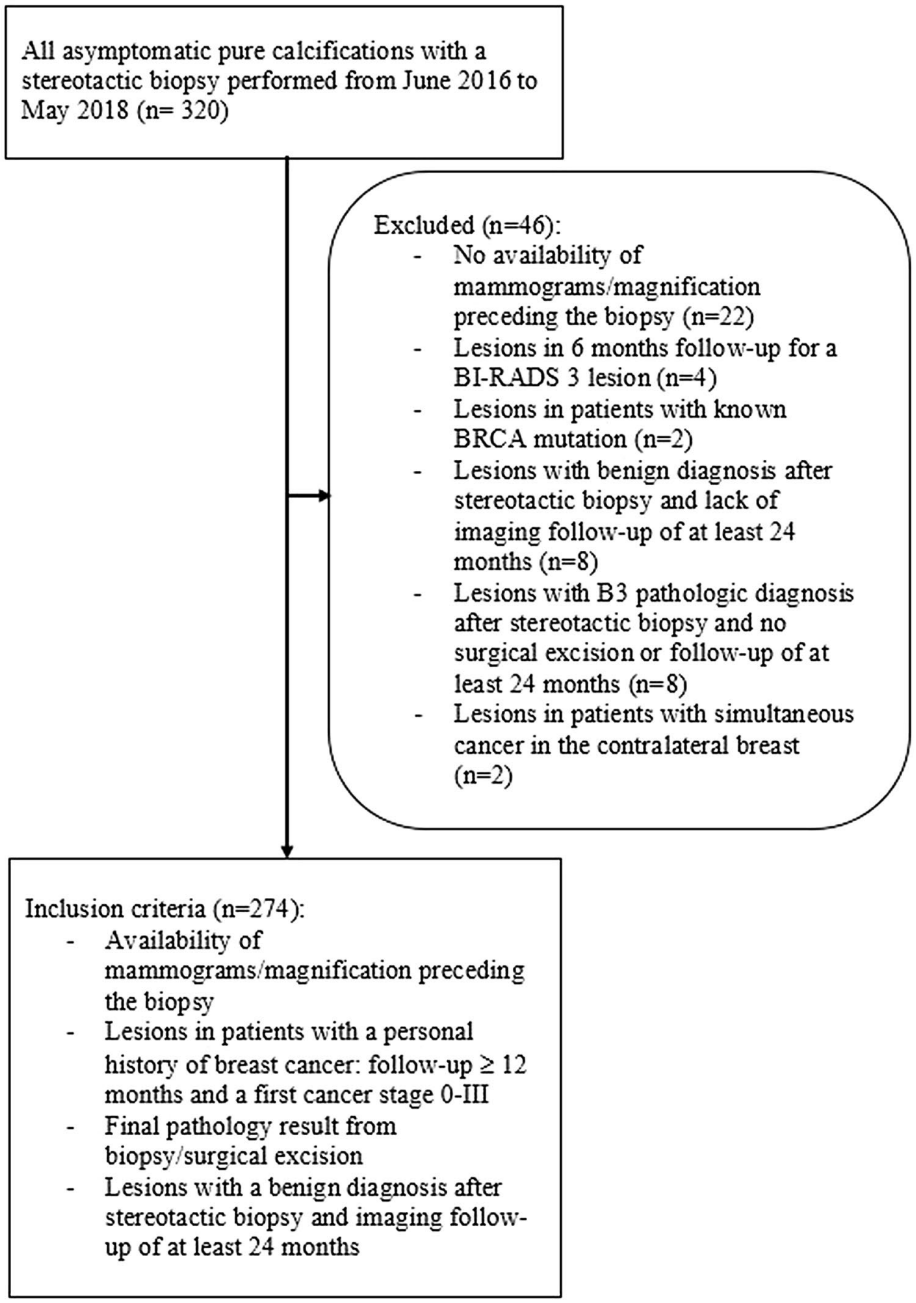
Flowchart of calcifications undergoing stereotactic biopsy and patients with and without a personal history of breast cancer

A flowchart of the cases included for analysis is shown in Fig. 1. A total of 268 women (median age, 54 years [interquartile range (IQR), 50–65 years]) with 274 lesions were included in this study. A PHBC was present in 46 of 268 (17.1%) patients (61 years, [IQR, 54–71 years]), and no PHBC was present in 222 of 268 (82.9%) patients (54 years, [IQR, 49–64 years]) (*P* = 0.0002). In patients with a PHBC, the median age was not significantly different between the subgroup with newly diagnosed malignancy (60.5 years, [IQR, 54–71 years]) and the subgroup with a benign histologic diagnosis (62.5 years, [IQR, 54–71 years](*P* = 0.881). Similarly, in patients without a PHBC, the median age was not significantly different between the subgroup with newly diagnosed malignancy (54 years, [IQR, 54–71 years]) and the subgroup with a benign histologic diagnosis (53 years, [IQR, 50–67 years](*P* = 0.881).

In patients without a PHBC mammography was performed as prevalent screening in 31 of 222 (14.0%) cases and as incident screening in 191 of 222 (86.0%) cases. A biopsy was performed bilaterally in 6 of 222 (2.7%) cases. A previous history of surgical excision for other than malignant lesions was present in 14 of 222 (6.3%) of patients, and the stereotactic biopsy was performed in the contralateral and in the ipsilateral breast in 4 of 14 (28.6%) and 10 of 14 (71.4%) cases, respectively.

In patients with a PHBC, 2 of 46 (4.3%) had a previous synchronous bilateral carcinoma, 3 of 46 (6.5%) had a history of bilateral breast cancer in 10-, 13- and 18-years’ time interval between the two cancers, and 1 of 46 (2.2%) had a history of local recurrence after 5 years from the first breast cancer diagnosis. Mastectomy was performed in 10 of 46 (21.7%) patients. In 22 (47.8%) cases, calcifications were also present at previous cancer diagnosis; in 18 (39.2%) cases no calcifications were present, whereas in 6 (13.0%) cases no information regarding imaging features of previous cancer was available.

Pathology results after either stereotactic biopsy or subsequent surgical excision are reported in Table 1. DCIS (67 of 274 lesions, 24.5%) was overall the most frequent diagnosis followed by atypical ductal hyperplasia (64 of 274 lesions, 23.3%). Sclerosing adenosis (12 of 46 lesions, 17.5%) was the most frequent diagnosis in women with a PHBC followed by DCIS and usual ductal hyperplasia (both 10 of 46 lesions, 21.7%). Atypical ductal hyperplasia (60 of 228 lesions, 26.3%) was the most frequent diagnosis in women without a PHBC followed by DCIS (57 of 228 lesions, 25%).

**Table 1:** Final pathologic diagnosis after percutaneous stereotactic biopsy or surgical excision

Pathology at diagnosis	All lesions (*n* = 274)	Lesions in patients with a personal history of breast cancer (*n* = 46)	Lesions in patients without a personal history of breast cancer (*n* = 228)	*P* value
All lesions				.068
Sclerosing adenosis	48 (17.5)	12 (26.1)	36 (15.8)	.089
Usual ductal hyperplasia	44 (16.1)	10 (21.7)	34 (14.9)	.230
Atypical ductal hyperplasia	64 (23.3)	4 (8.7)	60 (26.3)	.009
Lobular neoplasia	2 (0.7)	0 (0)	2 (0.9)	.548
Papilloma	11 (4.0)	0 (0)	11 (4.8)	.133
Fibroadenoma	18 (6.6)	4 (8.7)	14 (6.1)	.548
Chronic inflammation/fibrosis	10 (3.6)	4 (8.7)	6 (2.6)	.045
DCIS Low grade Intermediate grade High grade DCIS with microinvasion	67 (24.5) 23 (34.3) 15 (22.4) 18 (26.9) 11 (16.4)	10 (21.7) 3 (30) 1 (10) 4 (40) 2 (20)	57 (25.0) 20 (37.7) 14 (26.4) 14 (26.4) 9 (17.0)	.617
IDC	9 (3.3)	2 (4.3)	7 (3.1)	.689
ILC	1 (0.4)	0 (0)	1 (0.4)	.689

Data in parentheses are percentages. DCIS = ductal carcinoma in situ, IDC = invasive ductal carcinoma, ILC = invasive lobular carcinoma

### Imaging findings and BI-RADS categorization

Calcifications were new compared to a previous examination in 92 of 274 (33.6%) cases, 14 of 46 (30.4%) patients with a PHBC and 78 of 228 (34.2%) patients without a PHBC. Calcifications were increasing in number compared to a previous examination in 137 of 274 (50%) cases, 30 of 46 (65.2%) patients with a PHBC and 107 of 228 (46.9%) patients without a PHBC. No previous examinations were available for comparison in 45 of 274 (16.4%) cases, 2 of 46 (4.4%) patients with a PHBC and 43 of 228 (18.9%) patients without a PHBC. 

Morphology and distribution of calcifications as well as the corresponding PPV are reported in Table 2. Calcifications classified according to morphology and distribution had a similar proportion of cases in patients with or without a PHBC (*P *= .917 and *P *= .686, respectively). The PPV of morphology and distribution in patients with or without a PHBC was similar (*P *= .434 and .089, respectively).

**Table 2:** Morphology and distribution of calcifications

Features	Lesions in patients with a personal history of breast cancer (*n* = 46)				Lesions in patients without a personal history of breast cancer (*n* = 228)				*P* value
	Cancer (*n* = 12)	No Cancer (*n* = 34)	Total (*n* = 46)	PPV (%)	Cancer (*n* = 65)	No Cancer (*n* = 163)	Total (*n* = 228)	PPV (%)	
Morphology									.144
Typically benign	0(0)	16(47.1)	16(34.8)	0	0(0)	40(24.5)	40(17.5)	0	
Amorphous	1(8.3)	2(5.9)	3(6.5)	33.3	4(6.2)	15(9.2)	19(8.3)	21.1	
Punctate	0(0)	1(2.9)	1(2.2)	0	1(1.5)	10(6.1)	11(4.8)	9.0	
Coarse heterogeneous	1(8.3)	2(5.9)	3(6.5)	33.3	5(7.7)	15(9.2)	20(8.9)	25.0	
Fine pleomorphic	7(58.4)	12(35.3)	19(41.3)	36.8	48(73.8)	80(49.1)	128(56.1)	37.5	
Fine linear/fine linear branching	3(25.0)	1(2.9)	4(8.7)	75.0	7(10.8)	3(1.9)	10(4.4)	70.0	
Distribution	*n* = 12	*n* = 18	*n* = 30		*n* = 65	*n* = 123	*n* = 188		.068
Diffuse	0(0)	0(0)	0(0)	0	0(0)	5(4.1)	5(2.7)	0	
Regional	1(8.3)	3(16.7)	4(13.3)	25	6(9.2)	21(17.1)	27(14.3)	22.2	
Grouped	9(75)	14(77.8)	23(76.7)	39.1	43(66.1)	80(65.0)	123(65.4)	35.0	
Linear	1(8.3)	0(0)	1(3.3)	100	5(7.8)	1(0.8)	6(3.2)	83.3	
Segmental	1(8.3)	1(5.5)	2(6.7)	50	11(16.9)	16(13.0)	27(14.4)	40.7	

Data in parentheses are percentages. PPV = positive predictive value

**Fig. 2:**
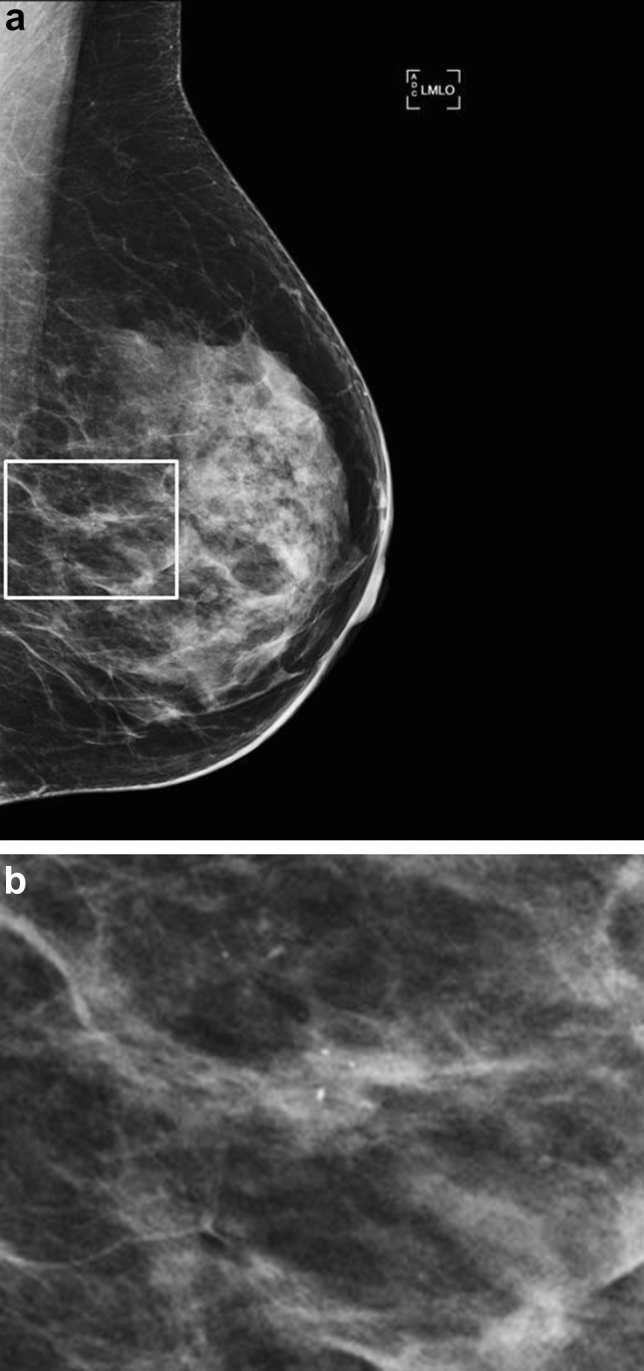
Mediolateral oblique mammogram (**a**) and corresponding magnification (**b**) of a 54-year-old woman without a personal history of breast cancer. Compared to a previous examination, new punctate calcifications (three calcifications, rectangle in A) were detected in the prepectoral region and classified as BI-RADS 3. Calcifications corresponded to low-grade ductal carcinoma in situ

Overall PPVs were the following: BI-RADS 2, 0% (0 of 56); BI-RADS 3, 9.1% (1 of 11, Fig. 2); BI-RADS 4a, 16.2% (6 of 37); BI-RADS 4b, 37.5% (48 of 128); BI-RADS 4c, 47.3% (18 of 38); and BI-RADS 5, 100% (4 of 4). The PPV of BI-RADS categories was similar in patients with and without a PHBC (*P *= .715, Table 3). Considering all lesions with risk of malignancy >2% (BI-RADS 4 and 5 category) the PPV was 36.7% (76 malignancy of 207 cases) overall, 40% (12 malignancy out of 30 cases) in patients with a PHBC and 36.1% (64 malignancy out of 177 cases) in patients without a PHBC (*P *= .687). Considering the dataset from the original read-out of calcifications which underwent stereotactic biopsy (i.e., including BI-RADS category 2 and 3), the PPV^1^ was 28.1% (77 malignancy out of 274 lesions) overall, 26.1% (12 malignancy out of 46 cases) in patients with a PHBC and 28.5% (65 malignancy out of 228 cases) in patients without a PHBC (*P*=.858).

**Table 3:** Distribution of the BI-RADS categories in patients with and without personal history of breast cancer

BI-RADS categories	Lesions in patients with a personal history of breast cancer (*n* = 46)				Lesions in patients without a personal history of breast cancer (*n* = 228)				*P* value
	Cancer (*n* = 12)	No cancer (*n* = 34)	Total (*n* = 46)	PPV (%)	Cancer (*n* = 65)	No cancer (*n* = 163)	Total (*n* = 228)	PPV (%)	.715
2	0(0)	16(47.1)	16(34.8)	0	0(0)	40(24.5)	40(17.5)	0	
3	0(0)	0(0)	0(0)	0	1(1.5)	10(6.1)	11 (4.8)	9.1	
4a	1(8.3)	4(11.8)	5(10.9)	20	5(7.7)	27(16.6)	32(14.1)	15.6	
4b	8(66.8)	13(38.2)	21(45.7)	38	40(61.5)	67(41.2)	107(46.9)	37.4	
4c	1(8.3)	1(2.9)	2(4.3)	50	17(26.1)	19(11.6)	36(15.8)	47.2	
5	2(16.6)	0(0)	2(4.3)	100	2(3.2)	0(0)	2(0.9)	100	

Data in parentheses are percentages. PPV = positive predictive value

Among patients with a PHBC 16 of 46 lesions (34.8%) underwent biopsy for BIRADS 2 calcifications compared to 42 of 228 lesions (18.4%) patients without a PHBC (*P*=.013). 

### Patients with personal history of breast cancer

Characteristics of women with a PHBC are reported in Table 4. In 27 of 46 (58.7%) cases, biopsy was performed in the ipsilateral and in 19 of 46 (41.3%) in the contralateral breast compared to the first breast cancer. A malignancy was diagnosed in the contralateral breast in 5 of 12 (41.7%) patients and in the ipsilateral breast in 7 of 12 (58.3%) patients. Only 1 of 12 (8.3%) patients had previous bilateral cancer.

**Table 4:** Characteristics of women with a personal history of breast cancer

Number of patients	Malignant calcifications	Benign calcifications	Total	*P* value
Time since first breast cancer diagnosis (years)*	*n* = 12	*n* = 34	*n* = 46	.026
1–2	1 (8.3)	3 (8.9)	4 (8.7)	.920
2–5	2 (16.7)	8 (23.5)	10 (21.7)	.617
5–10	0 (0)	13 (38.2)	13 (28.3)	.012
> 10	9 (75)	10 (29.4)	19 (41.3)	.005
Characteristic previous cancer**	*n* = 13	*n* = 38	*n* = 51	
Histologic type				.460
Ductal carcinoma in situ	1 (7.7)	9 (23.7)	10 (19.6)	.194
Invasive ductal	9 (69.2)	24 (63.1)	33 (64.8)	.689
Invasive lobular	1 (7.7)	3 (7.9)	4 (7.8)	1
Invasive other	2 (15.4)	2 (5.3)	4 (7.8)	.230
Regional lymph nodes status				.638
Negative	11 (84.6)	34 (89.5)	45 (88.2)	
Positive	2 (15.4)	4 (10.5)	6 (11.8)	
Cancer stage according to TNM				.644
0	1 (7.7)	9 (23.7)	10 (19.6)	.194
I	4 (30.8)	11 (28.9)	15 (29.4)	.920
II	7 (53.8)	16 (42.1)	23 (45.1)	.484
III	1 (7.7)	2 (5.3)	3 (5.9)	.764
Presence of calcifications				.269
Yes	6 (50.0)	16 (47.1)	22 (47.8)	
No	6 (50.0)	12 (35.3)	18 (39.2)	
Unknown	0 (0)	6 (17.6)	6 (13.0)	
Therapy first cancer**				
Surgical method				.253
Breast conserving surgery	9 (69.2)	32 (84.2)	41 (80.4)	
Mastectomy	4 (30.8)	6 (15.8)	10 (19.6)	
Adjuvant radiation therapy				.662
Yes	12 (92.3)	32 (84.2)	44 (86.3)	
No	1 (7.7)	6 (15.8)	7 (13.7)	
Adjuvant chemotherapy				.730
Yes	5 (38.5)	11 (28.9)	16 (31.4)	
No	8 (61.5)	27 (71.1)	35 (68.6)	
Endocrine therapy				.024
Yes	4 (30.7)	26 (68.4)	30 (58.8)	
Ongoing	2 (50)	16 (61.5)		
Terminated	2 (50)	10 (38.5)		
No	9 (69.2)	12 (31.6)	21 (41.2)	

Data in parentheses are percentages

*In case of bilateral metachronous breast cancer, first diagnosed cancer is considered here

**In case of bilateral metachronous breast cancer, both cancers have been considered separately

**Fig. 3:**
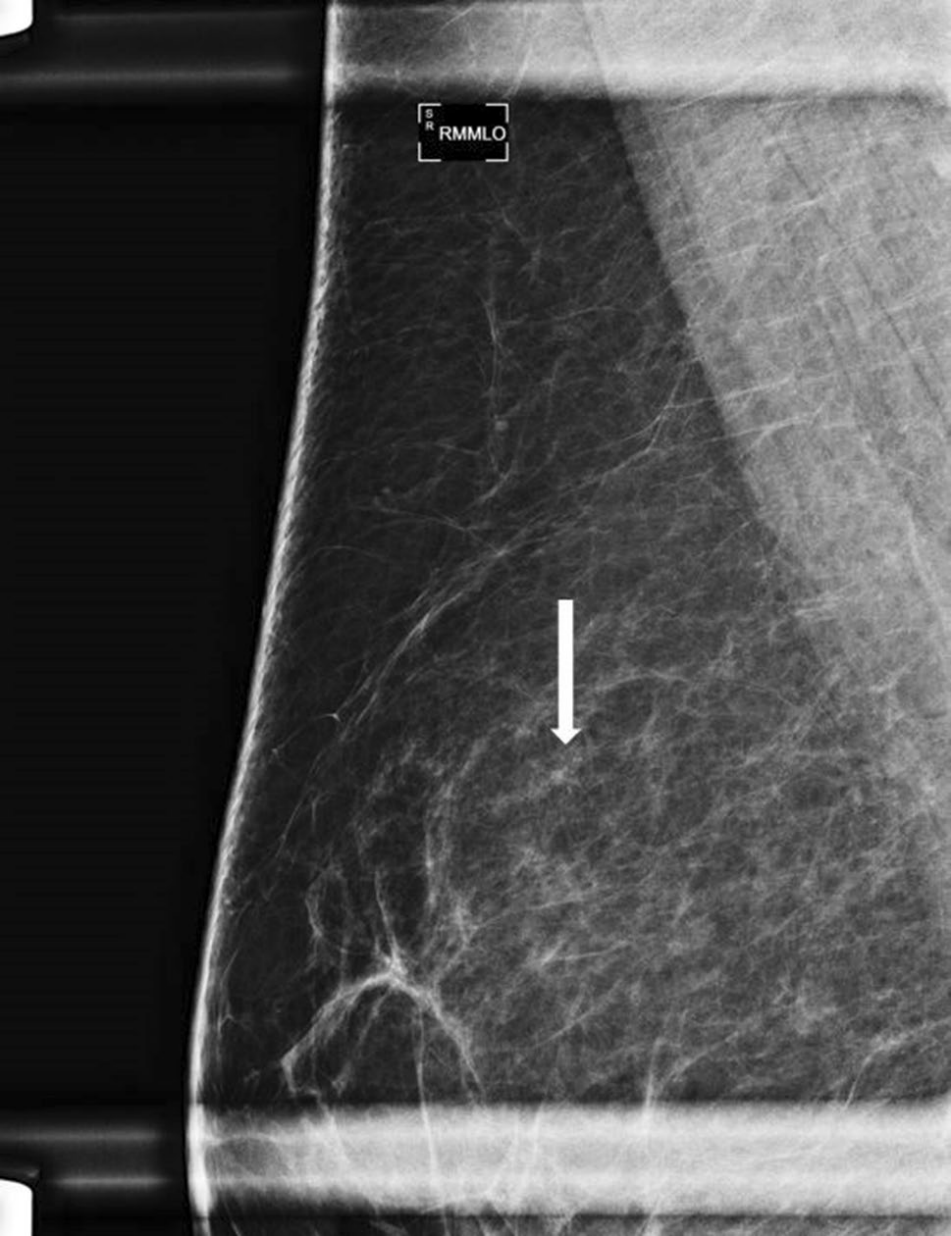
Magnification view (mediolateral oblique projection) of the upper quadrants of the right breast in a 55-year-old woman undergoing follow-up mammography one year after breast conserving surgery for invasive ductal carcinoma in the contralateral breast. Compared to a previous examination, new fine pleomorphic grouped calcifications (white arrow) were detected in the upper outer quadrant of the right breast and classified as BI-RADS 4b. Calcifications corresponded to usual ductal hyperplasia

**Fig. 4:**
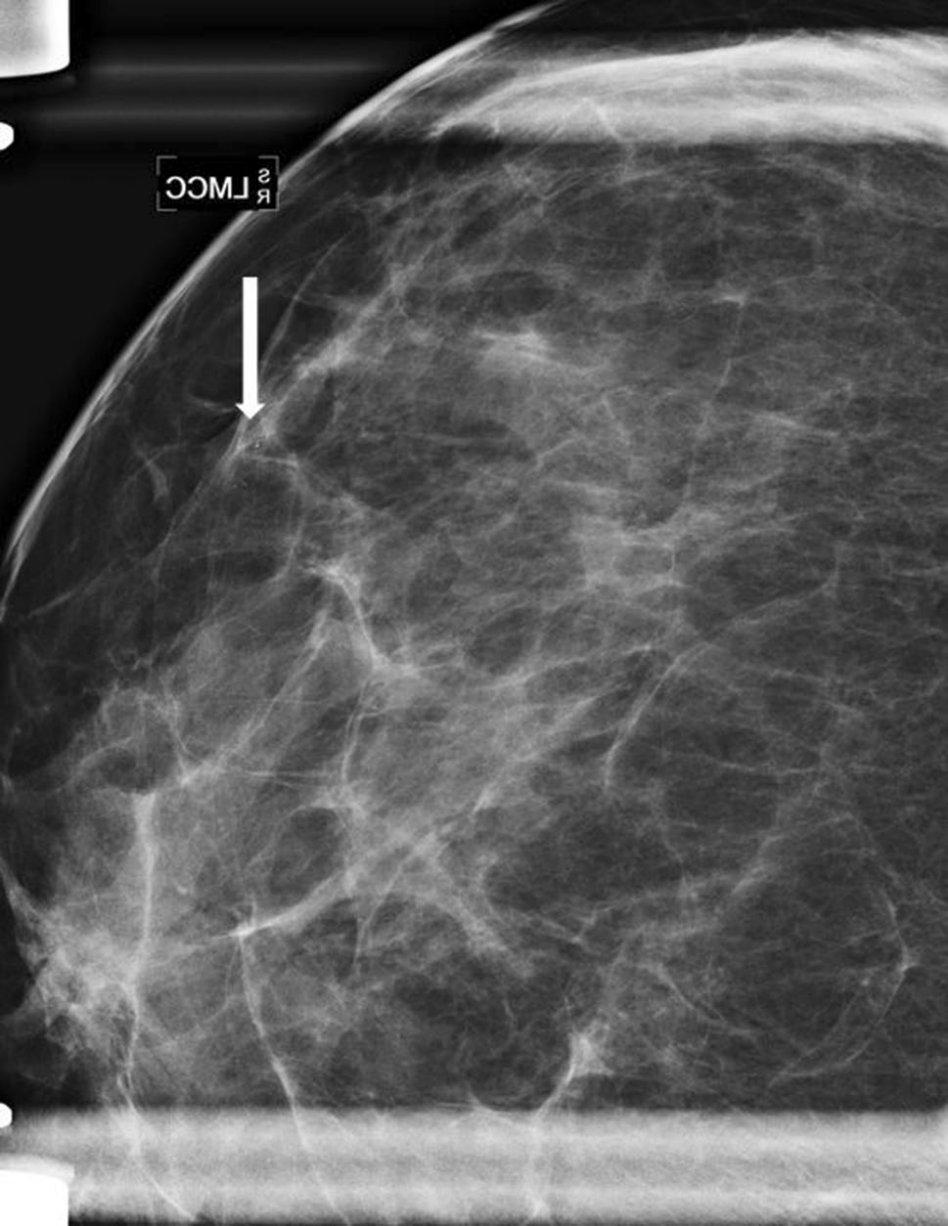
Magnification view (craniocaudal projection) of the outer left breast in a 63-year-old woman undergoing follow-up mammography one year after breast conserving surgery for invasive lobular carcinoma in the contralateral breast. Compared to a previous examination, new fine pleomorphic grouped calcifications (white arrow) were detected in the upper outer quadrant of the left breast and classified as BI-RADS 4b. Calcifications corresponded to high-grade ductal carcinoma in situ

In patients with previous breast cancer diagnosis 1–2 years before biopsy, only one contralateral malignancy was found (25%, 1 of 4 cases, Figs. 3 and 4). In patients with a previous breast cancer diagnosis 2–5 years before the biopsy, calcifications were malignant in 2 of 10 cases (20%) with one malignancy in the ipsilateral breast (10.0%) and one malignancy in the contralateral breast (10.0%). In patients with a previous breast cancer diagnosis 5–10 years before the biopsy, no calcifications were malignant (0 of 13 cases, 0%). In patients with a previous cancer diagnosis over 10 years before, calcifications were malignant in 9 of 19 (47.3%) cases, 6 of 9 (66.7%) in the ipsilateral breast and in 3 of 9 (33.3%) in the contralateral breast. No differences were observed in patients with benign and malignant calcifications diagnosis comparing the characteristics of previous breast cancer and the therapy approach (*P* > 0.024).

## Discussion

BI-RADS classification of breast calcifications is based on specific imaging features and should be applied regardless of personal factors such as a personal history of breast cancer (PHBC). Nevertheless, evaluation of calcifications may be biased when a PHBC is known. Our study showed a similar PPV for each BI-RADS category in patients with and without PHBC (36.7% vs. 40%; *P *= .687). Comparing calcification assessment blinded to the patient history to the original evaluation in the diagnostic setting, we found that a greater percentage of cases with BI-RADS 2 calcifications has undergone biopsy in the group of patients with a PHBC (34.8%) compared to the group without a PHBC (18.4%, *P *= .013). These data indicate that unnecessary biopsies may be partly avoided when calcification evaluation is based on their morphology, distribution and increase over time without accounting for a PHBC.

Assessment of breast calcifications can be challenging. Previous studies have reported a PPV of less than 30% for calcification classification [16, 20–22]. Moreover, a fair to moderate agreement among multiple readers is described in case of calcification assessment based on morphology and distribution [23–25]. To improve the accuracy of calcification evaluation, in our study all images were analyzed in consensus by two radiologists specialized in breast imaging and specifications for calcification assessment according to BI-RADS were provided. Considering all lesions with a risk of malignancy > 2%, we found a PPV of 36.7% proving that a strict use of BI-RADS classification can be used to improve accuracy in calcification assessment [26].

From BI-RADS category 2–5, a progressive PPV was found in both groups of patients. A PPV of 9.1% for lesions categorized BI-RADS 3 was higher than that commonly reported, and it can be explained by the low number of cases included in this category with only one case of malignancy, i.e., low-grade DCIS (13). BI-RADS category 4 encompasses a wide range of malignancy, and use of subcategories is suggested in order to provide a more detailed risk stratification assessment, a more accurate radiologic–pathologic correlation and ultimately support patient treatment decisions. Nevertheless, BI-RADS 4 subcategories remain scarcely applied, particularly in calcification assessment despite the proven advantages in terms of risk assessment [14, 16, 27]. In our study, we found a progressive risk of malignancy from category 4a to c, although with a slightly higher percentage for category 4a (16.2%) and a slightly lower percentage for category 4c (47.3%) compared to the corresponding expected ranges (2–10% and 51–95%). The PPV of morphology and distribution categories were similar in patients with or without PHBC (*P* = 0.144 and *P* = 0.068, respectively).

In patients with and without a PHBC, DCIS was similarly the most frequent diagnosis of malignancy (21.7% and 25.0%, respectively). The percentage of cases with ADH was higher in patients without a PHBC than in patients with a PHBC (26.3% and 8.7%, respectively). Although antihormonal therapy (e.g., tamoxifen and aromatase inhibitor) seems to reduce the incidence of clinically detected benign breast disease and could in part explain this finding, we found that a similar percentage of other benign lesions was present in the two groups of patients [28].

In patients with a PHBC of more than 10 years about 47% of the biopsied calcifications were malignant compared to 12.5% in case of breast cancer in 1–10 years before the biopsy. A malignancy was diagnosed in the contralateral breast in 41.7% patients and in the ipsilateral breast in 58.3% patients. Previous works reported that tumor recurrences at the original tumor site are in observed at a rate up to 2.5% per year between 2 and 6 years after treatment and are in general due to local treatment failure [29, 30]. Local imaging findings in the first two years after treatment usually represent benign lesions and cancer diagnosed after 10 years are usually new malignancies in a different site or in the contralateral breast [31]. A number of factors including breast cancer subtype, breast cancer treatment and stage are known to be associated with local recurrence. In our study, these factors did not differ between women with malignant and benign calcifications, but this could be also due to the limited number of cases included for each category [32].

Our study has some limitations. First, a single projection or magnification was provided for the calcification evaluation and no previous examinations were available for comparison. Although the growth rate could be not directly assessed, information regarding calcification increase over time was provided to increase the accuracy in the calcification assessment [33]. Second, in case of ipsilateral calcifications in patients with a PHBC, the presence of postoperative changes could have biased the evaluation. Nevertheless, fourteen patients without a PHBC and previous breast surgical excision have also been included. Third, the study was performed in an oncology referral center with a high proportion of patients in follow-up after breast cancer treatment and our results could may not be generalizable to other diagnostic settings. Fourth, family history was not considered owing to partly unavailable information. Finally, a limited number of cases were included and we are aware that a matched-population-based study would have strengthened the significance of our findings.

In conclusion, our study demonstrates that the PPV of calcifications is similar in women with or without PHBC when BI-RADS classification is applied. Calcification assessment should be based only on their morphology, distribution and increase over time. In case of suspicious calcifications in patients with a PHBC longer than 10 years we observed a higher risk of malignancy.

## Supplementary Information

Below is the link to the electronic supplementary material.

Supplementary file1 (DOCX 13 KB)

## Abbreviations

BI-RADS: Breast Imaging-Reporting and Data System
PHBC: Personal History of Breast Cancer
PPV: Positive predictive value
