# Supplementary material for: Mammographic calcifications undergoing percutaneous biopsy: outcome in women with and without a personal history of breast cancer
Source: Radiol Med. 2023 Jan 4;128(2):149–59. doi: 10.1007/s11547-022-01583-5 (PMC9938807; doi:10.1007/s11547-022-01583-5)
Supplement: Supplementary file 1 — Supplementary file1 (DOCX 13 KB) [file 11547_2022_1583_MOESM1_ESM.docx]

**Supplemental materials.** Calcification features and corresponding BI-RADS category.

| BI-RADS category | Calcification features |
| --- | --- |
| BI-RADS 2 | - Skin and vascular calcifications - Coarse (“popcorn-like”) calcifications - Large rod-like calcifications - Diffuse and small (<1 mm) round calcifications - Rim calcifications - Dystrophic calcifications - Milk of calcium |
| BI-RADS 3 | - Solitary group of punctate (<0.5 mm) calcifications in women without available previous mammograms for comparison |
| BI-RADS 4a | - New group of round calcifications or increasing, linear or segmental in distribution |
| BI-RADS 4b | - Grouped amorphous or fine pleomorphic calcifications - Grouped coarse heterogeneous calcifications |
| BI-RADS 4c | - Amorphous or fine pleomorphic calcifications with segmental or linear distribution - Fine linear or fine-linear branching calcifications regardless of their distribution |
| BI-RADS 5 | - Fine linear and branching calcifications with segmental distribution |
